# Understanding Dysregulated Behaviors and Compulsions: An Extension of the Emotional Cascade Model and the Mediating Role of Intrusive Thoughts

**DOI:** 10.3389/fpsyg.2016.00994

**Published:** 2016-06-29

**Authors:** Stefanie M. Jungmann, Noelle Vollmer, Edward A. Selby, Michael Witthöft

**Affiliations:** ^1^Department of Clinical Psychology and Psychotherapy, Johannes Gutenberg University of MainzMainz, Germany; ^2^Hospital for Psychiatry, Psychotherapy, and Psychosomatics, Fachklinik KatzenelnbogenKatzenelnbogen, Germany; ^3^Department of Psychology, Rutgers UniversityPiscataway, NJ, USA; ^4^Department of Clinical Psychology, Psychotherapy, and Experimental Psychopathology, Johannes Gutenberg University of MainzMainz, Germany

**Keywords:** behavioral dysregulation, compulsions, emotional cascades, (cognitive) emotion regulation, intrusion

## Abstract

**Objective:** The Emotional Cascade Model (ECM) by Selby et al. (2008) proposes that people often engage in dysregulated behaviors to end extreme, aversive emotional states triggered by a self-perpetuating vicious cycle of (excessive) rumination, negative affect, and attempts to suppress negative thoughts.

**Method:** Besides replicating the ECM, we introduced intrusions as a mediator between rumination and behavioral dysregulation and tested this extended ECM for compulsions as part of obsessive–compulsive disorders. A structural equation modeling approach was used to test this in a sample of *N* = 414, randomly recruited from the general population.

**Results:** Intrusions were found to fully mediate the effect of rumination on a broad array of dysregulated behaviors and compulsions. This mediation endured when controlling for symptoms of depression.

**Conclusion:** These findings support the idea that rumination fuels intrusions, which in turn foster dysregulated behaviors. Therefore, addressing rumination as well as intrusions may improve psychotherapeutic interventions for mental disorders characterized by dysregulated behaviors and/or extreme aversive emotional states.

## Introduction

The Emotional Cascade Model (ECM) by Selby et al. (2008) proposes that the relationship between aversive emotions and behavioral dysregulation is explained by a self-perpetuating cycle of rumination, negative thoughts, and negative affect. Excessive rumination leads to a strong negative affective state, in which negative emotional stimuli attract attention and increase rumination, which in turn progressively exacerbates negative affect (Selby et al., 2014). This emotional cascade results in extreme, aversive emotions and weakens the ability to turn one’s attention away from it. Finally, these emotions can be overwhelming, and it may be difficult to interrupt this cycle by functional and harmless means (e.g., reappraisal and distraction). Instead, people may use intensive types of behavioral emotion regulation (ER) to distract from rumination, many of which are harmful.

Behavioral dysregulation, which refers to behavioral ER strategies that are harmful, can include (but are not limited to): drinking alcohol to cope with problems, binge eating, extreme social reassurance seeking, and non-suicidal self-injuries (NSSI). Such behaviors are frequently discussed as a means of emotional avoidance (Tice et al., 2001; Chapman et al., 2006; Kingston et al., 2010). These behaviors may serve ER functions by allowing people to shift their attention away from unpleasant emotional states and toward, for example, bodily sensations (e.g., NSSI), taste (e.g., binge eating, alcohol), and social support and reassurance. Unfortunately, this relief is only short-term. In the long term, such behaviors may even trigger new ECs by entailing negative consequences such as a guilty conscience, negative bodily states (e.g., after excessive alcohol intake), and social problems (e.g., because of excessive social reassurance seeking).

Previous studies have found that rumination is related to psychiatric disorders, such as depression, anxiety, and personality disorders, and more specifically with dysregulated behaviors such as NSSI, binge eating, and alcohol abuse (Garnefski et al., 2001; Bushman et al., 2005; D’Acremont and Van der Linden, 2007; Garnefski and Kraaij, 2007; Nolen-Hoeksema et al., 2007; Aldao et al., 2010; Görgen et al., 2014, 2015). Selby et al. (2008, 2009), for example, have demonstrated that rumination is significantly correlated with drinking alcohol to cope, binge eating, social reassurance seeking, and urgency (the need to act impulsively and spontaneously when experiencing negative affect), both in borderline personality patients and in a non-clinical population, even when controlling for depressive symptoms. All four dysregulated behaviors formed one latent factor, suggesting that the ECM might be relevant to many types of dysregulated behaviors.

The ECM may explain why only some people experiencing negative life events or high levels of distress develop clinically relevant behavioral dysregulation: it can depend on their use of cognitive ER, specifically their tendency to habitually use rumination. Still the question remains why even people prone to rumination do not end up in an EC every time, and through which mechanisms rumination is associated with dysregulated behavior. Rumination is related to additional cognitive processes or strategies, such as intrusive thoughts, other blame, and attentional control (Garnefski et al., 2001; Santa Maria et al., 2012; Smets et al., 2012; Hsu et al., 2015). But intrusive thoughts in particular may play an especially pivotal role in the ECM. To stop ruminating, people often attempt to suppress their thoughts (Selby et al., 2008). Unfortunately, due to the rebound effect of thought suppression, this causes the opposite effect of frequent intrusive thoughts (Wegner et al., 1998).

Several studies have demonstrated that rumination contributes to intrusive thoughts (e.g., Santa Maria et al., 2012). Furthermore, intrusions can lead to dysfunctional behavior, such as avoidance in posttraumatic stress disorder (Horowitz, 1982), or can be a risk factor for self-harming behavior (Batey et al., 2010). These results suggest that intrusions (in terms of unwanted negative thoughts) might act as a mediator between rumination and the initiation of dysregulated behavioral responses proposed in the ECM. Some studies have investigated the mediating role of intrusive thoughts. A recent study found that intrusions mediate the relationship between trait anxiety and posttraumatic stress symptoms (Olatunji and Fan, 2015). Smets et al. (2012) showed the mediating role of intrusive thoughts in the association between rumination and depressive symptoms. To the best of our knowledge, intrusions as a mediator between rumination and the initiation of dysregulated behaviors, especially in the context of the ECM, have not been tested to date.

To be able to investigate this model it is important to clearly conceptualize and differentiate the constructs involved. Although the findings of previous studies are somewhat heterogeneous, multiple studies found evidence for a global construct of negative repetitive thoughts, instead of separate constructs such as rumination, catastrophizing, and worrying (e.g., Segerstrom et al., 2000). At first consideration one might tend to add intrusion to this concept and wonder how it might improve the assumptions of the ECM described above. Rumination is widely known and accepted as a (habitual) mental *strategy* that people unconsciously or consciously choose to deal with negative emotional information and feelings. It is a strategy of coping or emotion regulation, which can be influenced and altered directly, especially in psychotherapy (Dunn et al., 2013; Teismann et al., 2014).

Intrusions, in contrast, are an intermittent *symptom*. Therefore, people are unable to learn how to prevent their occurrence (as it is possible concerning rumination); they can only learn how to cope with and to appraise them. So intrusions may be comparable, to some extent, to other symptoms such as pain. Pain is highly correlated with rumination and catastrophizing, whereas rumination can trigger or intensify and focus one’s attention on pain and vice versa (Osman et al., 2000; Edwards et al., 2011). Considering these arguments, it appears reasonable to distinguish rumination and intrusions, because they represent distinct phenomena and may be related as described above. Research by Ciesla and Roberts (2002, 2007) supports this distinction.

Therefore, *the first aim of this study* was to replicate the ECM of Selby et al. (2008) in a large sample of the general population and to test whether intrusions mediate the association between rumination and behavioral dysregulation, even when controlling for current depressive symptoms. Therefore, similar to the original study by Selby et al. (2008), the latent rumination factor was created by the rumination and catastrophizing scales of the Cognitive Emotion Regulation Questionnaire (CERQ; Garnefski et al., 2001) and the rumination self and rumination symptoms scales of the Response Styles Questionnaire (for explanation, see Materials and Methods; Treynor et al., 2003) to enable a much broader and complete description of the construct. Because rumination is such a broad construct (Watkins, 2008), with regard to both the cognitive process and the cognitive content, we utilized multiple measures of rumination to create an encompassing latent variable for the construct of rumination. The CERQ scales assess the extent of thinking about feelings and (catastrophizing) thoughts when experiencing negative events in a more general manner, while the two scales of the RSQ focus on ruminative thinking about the self and one’s symptoms (e.g., fatigue and achiness). Regarding dysregulated behavior, we investigated the three above mentioned strategies drinking alcohol, seeking reassurance, symptoms of bulimia, and additionally urgency (i.e., acting impulsively). In order to replicate the findings of Selby et al. (2008) and for a better comparability of results, we used the same four dysregulated behavior variables.

The described indicators of dysregulated behavior were complemented by two more dimensions: the behavioral or reassurance-seeking scale of the Multidimensional Inventory of Hypochondriacal Traits (MIHT; Longley et al., 2005) and the Anger-Out scale of the State-Trait Anger Expression Inventory (STAXI; Spielberger, 1988). The behavioral scale of the MIHT assesses the tendency to seek medical reassurance and social support, which is supposed to reduce negative emotions and aversive emotional arousal in the short term, but to lead to decreased abilities to reassure oneself and to chronic anxiety and distress in the long term (Warwick and Salkovskis, 1990). This reassurance scale of the MIHT complements the previously investigated reassurance-seeking behavior. While the reassurance scale used by Selby et al. (2008) assesses the tendency to seek reassurance associated with self-doubt, the reassurance scale of the MIHT measures excessive reassurance seeking and social support for health concerns. The other three subscales of the MIHT (cognitive, perceptual, and affective scales) do not assess behaviors for regulating negative affect. The Anger-Out scale comprises verbal and behavioral expressions of anger and is considered to be the opposite of Anger-Control and Anger-In (being angry without showing it). Venting one’s anger is considered as an impulsive, emotion-driven, and mostly dysregulated behavior (Anestis et al., 2009). Both dimensions were found to be positively associated with rumination as well (Rusting and Nolen-Hoeksema, 1998; Martin and Dahlen, 2005; Ray et al., 2008; Dittmann, unpublished diploma thesis).

The previously described dysregulated behaviors occur in the context of many conditions, including substance abuse, personality disorders, or anxiety and affective disorders. In recent years, rumination as well as intrusions have been increasingly investigated in obsessive–compulsive disorders (OCD; Wahl et al., 2011a; García-Soriano et al., 2014; Moradi et al., 2014). Patients with OCD showed high levels of rumination and suffered from frequent intrusive thoughts (García-Soriano et al., 2014; Moradi et al., 2014). Especially in this mental disorder, intrusive thoughts are experienced as threatening, distressing, and uncontrollable (Abramowitz and Jacoby, 2015). Intrusive thoughts may be overwhelming and people may attempt to interrupt this aversive emotional state by dysfunctional behavior, in this case by compulsions. Comparable to other dysfunctional behaviors, as described above, compulsions can be conceptualized as an avoidance strategy that reduces aversive emotions in the short term, but leads to even more intrusions and negative emotions in the long term. In line with the ECM, compulsive behaviors may serve as a distraction mechanism to end or interrupt emotional cascades. So, OCD offers a good example to test the ECM as transdiagnostic model, because especially in this mental disorder the above described processes play a relevant role: ruminative thinking, intrusions, attempts to suppress distressing thoughts, negative affect, and compulsions as dysfunctional behavior. *The second aim of this study*, then, was to test whether the ECM and the postulated role of intrusions holds not only for behavioral dysregulation in this broad sense, but also for specific disorders in which intrusions play an important role. Therefore, we tested the expanded ECM (with intrusions as a mediator between rumination and dysregulated behavior) on compulsions.

## Materials and Methods

### Procedure and Participants

This study was part of a larger project investigating the relationship between ER and psychopathology in the general population. Questionnaires were sent to 3,000 adults randomly selected from the population registers of 10 German cities. They received all necessary information and signed a written declaration of consent. Participation was voluntary and unpaid. The study protocol adhered to the Declaration of Helsinki and was approved by the local ethics committee of the Psychological Institute.

Four hundred and twenty-three (14.1%) completed questionnaires were returned (which is acceptable considering the complexity of the series of measures; Diekmann, 1995). The data sets of nine participants had to be excluded due to completely missing subscales of at least one instrument. The final sample consisted of *N* = 414: The participants were 18-89 years old (*M* = 47.2 years; *SD* = 16.7; age ranges/distribution 18–27: 15%, 28–37: 17%, 38–47: 20%, 48–57: 21%, 58–67: 13%, 68–77: 11%, 78–89: 3%). Fifty-four percent were female (in the age ranges: 18–27 and 28–37 more women than men, *p* < 0.05), 31.7% were unmarried, 59.4% married, 8.4% divorced, or widowed. Only 1.9% reported to be without school graduation, 22.0% had finished secondary general school, 22.2% intermediate secondary school, 21.1% grammar school classes A-level, and 31.9% university. The level of education was not equivalent within the categories of age; age, and education showed a significant negative correlation (*r* = -0.25, *p* < 0.001). Women completed more often intermediate secondary school and grammar school classes A-level than men (*p* < 0.05).

Twenty-seven percent said they were suffering from a chronic or severe disease (e.g., asthma, neurodermatitis, or COPD). At the time of the inquiry, 21% of the sample were in psychotherapeutic or psychiatric treatment, 80% gave no reason for treatment, 6% stated a depressive disorder, 2% an anxiety disorder, 2% loss or posttraumatic stress disorder, 1% a somatoform disorder, 2% problems in the family/partnership, 1% problems at the workplace, and 4% other (e.g., tinnitus, eating disorder, or alcohol dependency).

The calculations of all test-retest reliabilities (**Tables 1** and **2**) were based on a follow-up study 7 months later. In this study, the same questionnaires were sent to all participants in the main study who had declared their willingness to participate again. One hundred and seventy-five people participated in this follow-up study (gender: 56.3% female; age: 19–85 years, *M* = 48.1 years, and *SD* = 16.7).

**Table 1:** Descriptive characteristics and latent correlations among ER, intrusions, and behavioral dysregulation.

Scale	Rum	Cata	RumSy	RumSe	Intrus	Reass	Cope	Out	Bulim	Behav	Urg
CERQ_Rum_											
CERQ_Cata_	0.67***										
RSQ_RumSy_	0.41***	0.48***									
RSQ_RumSe_	0.52***	0.44***	0.66***								
TSI_Intrus_	0.36***	0.51***	0.70***	0.53***							
DIRI_Reass_	0.34***	0.37**	0.41***	0.31**	0.43***						
DMQ_Cope_	0.17**	0.19**	0.24***	0.16**	0.36***	0.31***					
STAXI_Out_	0.09	0.15*	0.33***	0.13*	0.30***	0.17*	0.12				
EDI_Bulim_	0.25***	0.19**	0.31***	0.34***	0.35***	0.33**	0.26***	0.11			
MIHT_Behav_	0.31***	0.26***	0.22***	0.08	0.23***	0.34***	0.19**	0.30***	0.20***		
UPPS_Urg_	0.20**	0.36***	0.52***	0.42***	0.78***	0.46***	0.39***	0.44***	0.35***	0.30***	

*M*	2.79	1.93	1.93	1.90	2.00	1.67	1.37	1.75	1.36	2.69	2.89
*SD*	0.96	0.81	0.59	0.58	0.70	0.68	0.60	0.64	0.48	0.79	3.65
*Number of items*	3	3	8	7	5	2	5	3	7	8	5
α/*r*_tt_	0.66/0.78	0.73/0.84	0.84/	0.77/	0.72/0.76	0.47/0.55	0.82/0.91	0.81/0.66	0.77/0.84	0.83/0.79	0.74/0.82

*CERQ_*Rum*_/Rum = Rumination scale of the Cognitive Emotion Regulation Questionnaire (shortened German version); CERQ_*Cata*_/Cata = Catastrophizing Scale of the Cognitive Emotion Regulation Questionnaire (shortened German version); RSQ_*RumSy*_/RumSy = Rumination about Symptoms of the Response Styles Questionnaire; RSQ_*RumSe*_/RumSe = Rumination about Self of the Response Styles Questionnaire; TSI_*Intrus*_/Intrus = Intrusion scale of the Thought Suppression Inventory; DIRI_*Reass*_/Reass = Reassurance Seeking scale of the Depressive Interpersonal Relationships Inventory; DMQ_*Cope*_/Cope = Drinking to Cope scale of the Drinking Motives Questionnaire; STAXI_*Ou*_/Out = Anger Out scale of the State-Trait Anger Expression Inventory; EDI_*Bulim*_/Bulim = Bulimia scale of the Eating Disorder Inventory; MIHT_*Behav*_/Behav = Behavioral scale of the Multidimensional Inventory of Hypochondriacal Traits; UPPS_*Urg*_/Urg = Urgency scale of the Urgency, (Lack) of Premeditation, (Lack) of Perseverance, and Sensation Seeking Impulsive Behavior Scale; α = Cronbach’s alpha; *r_*tt*_* = Test-retest-reliability (over 7 months); *r*_*tt*_ not available for RSQ in this study; ^∗^*p* ≤ 0.05; ^∗∗^*p* ≤ 0.01; ^∗∗∗^*p* ≤ 0.001.*

**Table 2:** Descriptive characteristics and latent correlations among ER, intrusions, and compulsions.

Scale	Rum	Cata	RumSy	RumSe	Intrus	Hoard	Check	Order	Neutral	Wash
CERQ_Rum_										
CERQ_Cata_	0.67***									
RSQ_RumSy_	0.41***	0.49***								
RSQ_RumSe_	0.52***	0.44***	0.66***							
TSI_Intrus_	0.36***	0.51***	0.70***	0.53***						
OCI_Hoard_	0.08	0.18**	0.26***	0.15**	0.48***					
OCI_Check_	0.18**	0.31***	0.38***	0.23***	0.51***	0.58***				
OCI_Order_	0.10	0.28***	0.27***	0.25***	0.36***	0.40***	0.61***			
OCI_Neutral_	0.16*	0.39***	0.33***	0.16*	0.49***	0.57***	0.77***	0.73***		
OCI_Wash_	0.10	0.38***	0.22***	0.20**	0.36***	0.44***	0.55***	0.59***	0.75***	

*M*	a	a	a	a	a	0.89	0.82	1.12	0.48	0.39
*SD*	a	a	a	a	a	0.90	0.86	1.00	0.68	0.60
*Number of items*	a	a	a	a	a	3	3	3	3	3
α/*r*_tt_	a	a	a	a	a	0.83/	0.81/	0.86/	0.60/	0.65/

*CERQ_*Rum*_/Rum = Rumination scale of the Cognitive Emotion Regulation Questionnaire (shortened German version); CERQ_*Cata*_/Cata = Catastrophizing Scale of the Cognitive Emotion Regulation Questionnaire (shortened German version); RSQ_*RumSy*_/RumSy = Rumination about Symptoms of the Response Styles Questionnaire; RSQ_*RumSe*_/RumSe = Rumination about Self of the Response Styles Questionnaire; TSI_*Intrus*_/Intrus = Intrusion scale of the Thought Suppression Inventory; OCI_*Hoard*_/Hoard = Hoarding scale of the Obsessive–Compulsive Inventory; OCI_*Check*_/Check = Checking scale of the Obsessive–Compulsive Inventory; OCI_*Order*_/Order = Ordering scale of the Obsessive–Compulsive Inventory; OCI_*neutral*_/Neutral = Neutralizing scale of the Obsessive–Compulsive Inventory; OCI_*Wash*_/Wash = Washing scale of the Obsessive–Compulsive Inventory; α = Cronbach’s alpha; *r_*tt*_* = Test-retest-reliability (over 7 months); *r*_*tt*_ not available for OCI in this study. ^a^Descriptive characteristics see **Table 1**; ^∗^*p* ≤ 0.05; ^∗∗^*p* ≤ 0.01; ^∗∗∗^*p* ≤ 0.001.*

### Questionnaires

#### Cognitive Emotion Regulation Questionnaire (CERQ; Garnefski et al., 2001; German version: Loch et al., 2011)

The CERQ is a 36-item scale measuring nine dimensions of cognitive ER (see **Table 1** for scale descriptions, Cronbach’s α, and test–retest reliability). Each of the dimensions describes possible cognitive reactions to aversive events and related negative emotions. Items are coded on a five-point Likert scale ranging from 1 = *(almost) never* to 5 = *(almost) always*. Many international studies supported its good psychometric properties, with Cronbach’s α between 0.70 and 0.80 and good validity (e.g., D’Acremont and Van der Linden, 2007; Garnefski and Kraaij, 2007; Selby et al., 2008). In this study, we used the rumination (e.g., “I often think about how I feel about what I have experienced”) and catastrophizing (e.g., “I keep thinking about how terrible it is what I have experienced”) scales of the shortened German version (3 items per factor and 27 items in total).

#### Response Styles Questionnaire (RSQ, short version; Treynor et al., 2003; German version: Kühner et al., 2007)

The 23-item short version of the RSQ consists of the scales: rumination self/reflection (e.g., “When I feel depressed I analyze my personality to try to understand why I am depressed”), rumination symptoms/brooding (e.g., “When I feel depressed I think about my feelings of fatigue and achiness”), and distraction (e.g., “I go to a favorite place to get my mind off my feelings”). The respondents are asked how often (1 = *almost never* to 4 = *almost always*) they react to a depressive mood by using one of the three strategies. Acceptable to good reliability (α: 0.77–0.84) and validity were reported for both versions (English and German). Only the two rumination scales were used in the current study.

#### Thought Suppression Inventory (TSI; Rassin, 2003).

The TSI includes the dimensions intrusion, (successful) suppression, and suppression attempts. Each scale consists of five items. Respondents are asked to quote their degree of approval on a five-point Likert scale (1 = *strongly disagree* to 5 = *strongly agree*). In this study only the intrusion scale was used (“I have many unpleasant thoughts”; “I experience many emotions that are too intense to control”; “I have thoughts which I would rather not have”; “I regularly *hear* unexplainable things inside my head, such as my own voice, or the voices of people who are not present”; “I am unable to concentrate”). This scale showed an acceptable internal consistency in previous studies and good test-retest reliability (Rassin, 2003; Wismeijer, 2012).

#### Obsessive–Compulsive Inventory-Revised (OCI-R; Foa et al., 2002; German version: Gönner et al., 2007)

The OCI-R is a shortened version of the Obsessive–Compulsive Inventory that measures OCD symptoms. It consists of 18 items and provides scores of six subscales: washing, checking, ordering, obsessing, hoarding, and neutralizing (scale: 0–4). The psychometric properties of the OCI-R showed good to excellent internal consistency, test–retest reliability, and convergent validity in both clinical and non-clinical samples. We used the scales washing, checking, ordering, hoarding, and neutralizing (e.g., counting and repeating words) to compute a second-order factor of compulsions. The subscale “obsessing” was excluded because it does not assess compulsive behaviors or behaviors regulating negative affect (e.g., “I find it difficult to control my own thoughts”).

#### Multidimensional Inventory of Hypochondriacal Traits (MIHT; Longley et al., 2005; German version: Witthöft et al., 2010)

The MIHT comprises 31 items (five-point Likert scale: 1 = *strong disagreement* to 5 = *strong agreement*) for the four dimensions affective, cognitive, behavioral, and perceptual of the cognitive-behavioral model of health anxiety and hypochondriasis. Acceptable to good properties are reported (e.g., α ≥ 0.75). In the current study, only the behavioral subscale was used (assessing the tendency to seek social support and medical reassurance as a response to physical symptoms and signs of illness).

#### Depressive Interpersonal Relationships Inventory, Reassurance Seeking subscale (DIRI-RS; Joiner et al., 1992)

Consisting of four items, the DIRI-RS measures a person’s tendency to seek reassurance from others. The items are answered on a seven-point Likert scale (1 = *(almost) never* to 7 = *(almost) always*). In the current study only two items were used (“Do you frequently seek reassurance from the people you feel close to as to whether they *really* care about you?”, “Do the people you feel close to sometimes become irritated with you for seeking reassurance from them about whether they r*eally* care about you?”), because the other two were nearly identical. Past studies demonstrated good psychometric properties for the reassurance-seeking subscale (Joiner and Schmidt, 1998).

#### Eating Disorder Inventory-2 (EDI-2; Garner et al., 1983; German version: Paul and Thiel, 2004)

The EDI is a 64-item self-report questionnaire that measures the degree to which participants exhibit pathological eating behaviors. It consists of eight subscales, of which only the bulimia subscale was used. This scale assesses binge eating and purging with seven items coded on a six-point Likert scale (1 = *never* to 6 = *always*). Good reliabilities and validity were reported in previous studies (Garner et al., 1983; Paul and Thiel, 2004).

#### Drinking Motives Questionnaire-Revised (DMQ-R; Cooper et al., 1992; German version: Kuntsche et al., 2006)

The DMQ is a self-report measurement assessing how often people consume alcohol for several motives. The scale consists of the dimensions coping motives, enhancement motives, and social motives. Each dimension is measured by five items answered on a six-point Likert scale ranging from 1 = *never* to 6 = *almost always*. In this study only the Drinking to Cope scale was used, which measures the degree to which people drink alcohol to deal with problems and negative affect.

#### State-Trait Anger Expression Inventory (STAXI; Spielberger, 1988; German version: Schwenkmezger et al., 1992).

The STAXI allows the assessment of state and trait anger as well as several ways to express or not to express anger (Anger-Out, Anger-In, and Anger-Control). Participants express their agreement with 44 items on a four-point Likert scale (1 = *(almost) never* to 4 = *(almost) always*). For this study, only the three most selective items of the Anger-Out scale were used (due to the large number of questionnaires and the need to keep the survey short to encourage participation, some instruments had to be shortened).

#### UPPS Impulsive Behavior Scale (UPPS; Whiteside and Lynam, 2001; German version: Keye et al., 2009)

The UPPS, with 45 items (Brief German version: 20 items), asks about behaviors mirroring the scales Urgency, (lack of) Premeditation, (lack of) Perseverance, and Sensation Seeking. A five-point Likert scale is used (1 *= strong disagreement* to 5 = *strong agreement*). Good psychometric properties were reported (e.g., Cronbach’s α: 0.72–0.91; Whiteside and Lynam, 2001; Keye et al., 2009). In the current study, only the Urgency scale consisting of five items was used.

#### Patient Health Questionnaire (PHQ-9; Kroenke et al., 2001; German version: Gräfe et al., 2004)

The PHQ allows the screening, estimation of severity, and measurement of the progress of eight mental disorders. In this study, only the depression module (PHQ-9) was applied. The PHQ-9 assesses impairment due to depressive symptoms during the prior two weeks. Items are answered on a four-point Likert scale (1 = *never* to 4 = *every day*). The authors report evidence for good reliability (α = 0.88; *r*_tt_ = 0.81–0.96) and validity.

### Statistical Analyses

A structural equation modeling (SEM) approach was used to investigate the hypotheses. The SEM approach is advantageous in that the latent structures of constructs are explicitly modeled and critically evaluated according to model fit indices, and that relations between latent variables represent true score correlations that are independent of measurement error. In a first step, measurement models were computed for each latent factor (behavioral dysregulation, compulsions, rumination, and intrusions). Then, we tested the general models replicating the ECM (rumination and behavioral dysregulation/compulsions) and the models with mediation. Using SEM, we only tested the hypotheses. Other alternative analyses beyond compulsions were not conducted (i.e., no other symptoms as dependent variable). The analyses were performed using Mplus version 7.2^©^ (Muthén and Muthén, 1998–2014). Mplus enables the researcher to examine confirmatory models with categorical indicators modeled by a two-parameter normal-ogive IRT model, in an integrated and generalized approach for measurement and structural models with latent variables (Glöckner-Rist and Hoijtink, 2003). The analyses of the measurement models were conducted with the robust mean and variance adjusted weighted least squares (WLSMV) procedure, which is insensitive to non-normal distributions. The WLSMV procedure is based on tetrachoric correlations, which can be biased due to low cell frequencies. So, we collapsed rarely used response categories to obtain response frequencies of at least 5% in each cell (Brown and Benedetti, 1977). Missing values were replaced in Mplus 7.2^©^ via full information maximum likelihood estimation (FIML).

Using SEM instead of standard regression methods to test mediation can have some critical advantages. Gunzler et al. (2013) enumerate, for example: construction of latent variables, examination of complicated models in a single analysis, handling of missing data, and model fit information. Furthermore, in regression analyses each variable has to be categorized a priori as cause or effect. This may be problematic, especially in cross-sectional designs. Testing mediation, Mplus uses the terms direct and indirect effect and computes their significance.

As indices for model fit, the ratio of χ^2^/df (1:2, max. 1:3; Bentler, 2007), the RMSEA (Root Mean Square Error of Approximation), the CFI (Comparative Fit-Index) and the TLI (Tucker Lewis-Index) were chosen. The RMSEA represents acceptable model fit <0.10, good fit <0.08, and very good fit <0.05 (Browne and Cudeck, 1993). The TLI and CFI indicate acceptable fit >0.90 and good to very good fit >0.95 (Bentler, 2007).

## Results

### Descriptive Statistics and Interrelations among the Measures Used

Descriptive characteristics and latent relationships among ER, intrusions, and the six forms of dysregulated behaviors (reassurance of the DIRI, drinking to cope, anger-out, bulimia, social support/reassurance of the MIHT, and urgency) are presented in **Table 1**. Regarding compulsions (hoarding, checking, ordering, neutralizing, and washing), **Table 2** shows descriptive characteristics and latent correlations with ER and intrusions. Cronbach’s α values range between 0.60 (neutralizing scale of the OCI) and 0.86 (ordering scale of the OCI), and the test–retest reliability is between 0.66 (anger-out of the STAXI) and 0.91 (drinking to cope). The two items of the DIRI show lower values: α = 0.47 and *r*_tt_ = 0.55.

### Model Fit of the Measurement Models

First of all, the measurement models for each higher-order factor were tested. The model fits were: for behavioral dysregulation: χ^2^(399): 567.81 (*p* < 0.001), CFI: 0.97, TLI: 0.97, and RMSEA: 0.032; for compulsions: χ^2^(85): 158.86 (*p* < 0.001), CFI: 0.98, TLI: 0.98, and RMSEA: 0.046; for rumination: χ^2^(185): 676.28 (*p* < 0.001), CFI: 0.90, TLI: 0.89, and RMSEA: 0.080; and for intrusions: χ^2^(5): 14.83 (*p* < 0.001), CFI: 0.99, TLI: 0.98, RMSEA: 0.069.

### Replication of the ECM (General Models without mediation)

We tested two models: model 1, the ECM for dysregulated behavior in a broader range; and model 2, the ECM for compulsions. First, the general ECM models (model 1: rumination, intrusions, and behavioral dysregulation; model 2: rumination, intrusions, and compulsions) were calculated as SEMs to test whether they fit the data well using standard model fit criteria (Hu and Bentler, 1998) and whether the four ER dimensions and the six behavioral dysregulation scales/the five compulsion scales, form second-order factors. The fit for both models was adequate to good: **Model 1**: χ^2^(1471): 2102.15 (*p* < 0.001), CFI: 0.94, TLI: 0.93, and RMSEA: 0.032. In this model, rumination (considered as the latent factor of negative, ruminative ER) had a strong latent correlation with behavioral dysregulation (*r* = 0.68, *p* ≤ 0.001) and intrusions (*r* = 0.76, *p* ≤ 0.001). Intrusions and behavioral dysregulation also showed a strong latent association (*r* = 0.80, *p* ≤ 0.001). The fit for **model 2** was also good: χ^2^(767): 1275.31 (*p* < 0.001), CFI: 0.94, TLI: 0.94, and RMSEA: 0.040. Rumination showed medium-high latent relationships with compulsions (*r* = 0.43, *p* ≤ 0.001) and high relations with intrusions (*r* = 0.76, *p* ≤ 0.001). Intrusion was also strongly associated with compulsions (*r* = 0.57, *p* ≤ 0.001). All correlations were significant on the 0.1%-level.

### Testing the Mediational Effect of Intrusion on the Relationship between Rumination and Behavioral Dysregulation (Model 1)

**FIGURE 1:**
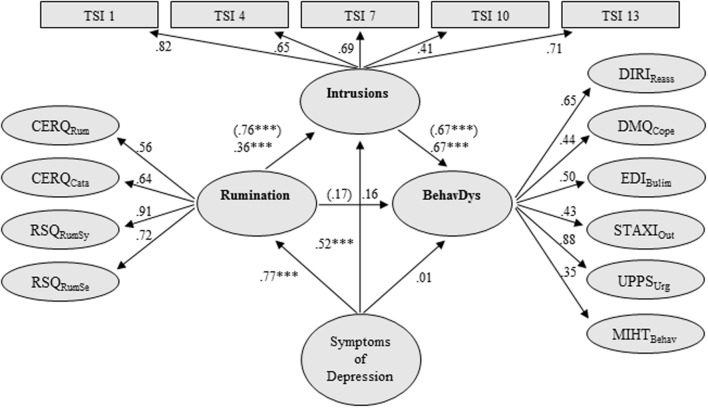
**Structural equation model (SEM) testing the mediational effect of intrusion on the relationship between rumination and behavioral dysregulation, controlling for symptoms of depression**. Rumination = Rumination latent variable; BehavDys = Behavioral Dysregulation latent variable; TSI = Thought Suppression Inventory; Intrusions = Intrusions latent variable; CERQ_Rum_ = Rumination scale of the Cognitive Emotion Regulation Questionnaire (shortened German version); CERQ_Cata_ = Catastrophizing Scale of the Cognitive Emotion Regulation Questionnaire (shortened German version); RSQ_RumSy_ = Rumination about Symptoms of the Response Styles Questionnaire; RSQ_RumSe_ = Rumination about Self of the Response Styles Questionnaire; DIRI_Reass_ = Reassurance Seeking scale of the Depressive Interpersonal Relationships Inventory; DMQ_Cope_ = Drinking to Cope scale of the Drinking Motives Questionnaire; EDI_Bulim_ = Bulimia scale of the Eating Disorder Inventory; STAXI_Out_ = Anger-Out scale of the State-Trait Anger Expression Inventory; UPPS_Urg_ = Urgency scale of the Urgency, Lack of Premeditation, Lack of Perseverance, Sensation Seeking-Impulsivity scale; MIHT_Behav_ = Behavioral scale of the Multidimensional Inventory of Hypochondriacal Traits; Symptoms of Depression were quantified by the 9 items of the PHQ-9 (factor loadings: 0.65–0.86); all scales are latent variables; single items are not presented due to space limitations; values in brackets: β-regression weights in SEM without control of depressive symptoms; all factor loadings were significant on the 1% level; ^∗∗∗^*p* ≤ 0.001; model fit: χ^2^(1999): 2790.02 (*p* < 0.001), CFI: 0.93, TLI: 0.93, and RMSEA: 0.031. *R*^2^ BehavDys: 0.65.

The factor loadings of each latent second-order variable on its corresponding variables and the regression paths between latent variables of model 1 are displayed in **Figure 1**. All indicators of dysregulated behaviors loaded substantially on their second-order factor. SEM analysis of this model, including control of depressive symptoms, indicated an adequate to good fit to the data (χ^2^(1999): 2790.02 (*p* < 0.001), CFI: 0.93, TLI: 0.93, and RMSEA: 0.031).

The effects of rumination no longer predicted behavioral dysregulation directly, after adding intrusion as a mediator (β = 0.17 and *p* = 0.13). This suggests that the effect of rumination on behavioral dysregulation was fully mediated by intrusion. Rumination was highly associated with intrusion (β = 0.76). Intrusion was also highly associated with behavioral dysregulation (β = 0.67). The indirect effect from rumination to dysregulated behavior was 0.51 (*p* < 0.001, 95% CI [0.338, 0.684]).

This meditational model remained significant after controlling for depressive symptoms (β = 0.36: rumination–intrusion; β = 0.67: intrusion–behavioral dysregulation; indirect effect from rumination to dysregulated behavior: 0.24, *p* < 0.001, 95% CI [0.116, 0.389]). Symptoms of depression were significantly associated with intrusion (β = 0.52) and rumination (β = 0.77), but not with behavioral dysregulation (β = 0.01; **Figure 1**). This model explained 65% of the variance of behavioral dysregulation, 14% more than the same model without intrusions as a mediator (*R*^2^ = 0.51; χ^2^(1697): 2413.75 (*p* < 0.001), CFI: 0.93, TLI: 0.93, and RMSEA: 0.032; using MLVM estimator).

### Testing the Mediational Effect of Intrusion on the Relationship between Rumination and Compulsions (Model 2)

**FIGURE 2:**
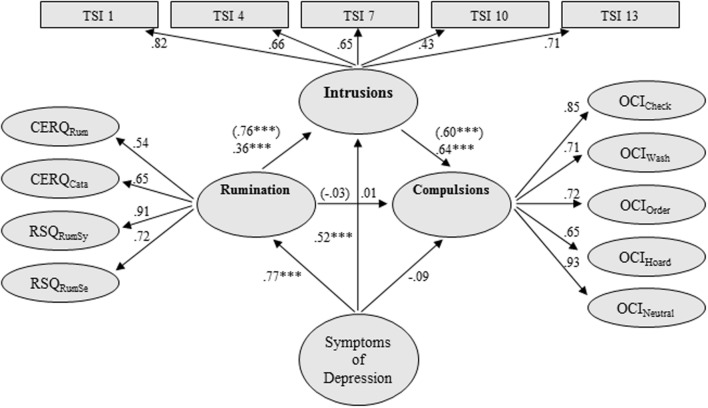
**Structural equation model (SEM) testing of the mediational effect of intrusions on the relationship between rumination and compulsion, controlling for symptoms of depression**. Rumination = Rumination latent variable; Compulsions = Compulsions latent variable; TSI = Thought Suppression Inventory; Intrusions = Intrusions latent variable; CERQ_Rum_ = Rumination scale of the Cognitive Emotion Regulation Questionnaire (shortened German version); CERQ_Cata_ = Catastrophizing Scale of the Cognitive Emotion Regulation Questionnaire (shortened German version); RSQ_RumSy_ = Rumination about Symptoms of the Response Styles Questionnaire; RSQ_RumSe_ = Rumination about Self of the Response Styles Questionnaire; OCI_Check_ = Checking scale of the Obsessive–Compulsive Inventory; OCI_Wash_ = Washing scale of the Obsessive–Compulsive Inventory; OCI_Order_ = Ordering scale of the Obsessive–Compulsive Inventory; OCI_Hoard_ = Hoarding scale of the Obsessive–Compulsive Inventory; OCI_Neutral_ = Neutralizing scale of the Obsessive–Compulsive Inventory; Symptoms of Depression were quantified by the 9 items of the PHQ-9 (factor loadings:0.65–0.86); all scales are latent variables; single items are not presented due to space limitations; values in brackets: β-regression weights in SEM without control of depressive symptoms; all factor loadings were significant on the 1% level; ^∗∗∗^*p* ≤ 0.001; model fit: χ^2^(1160): 1782.38 (*p* < 0.001), CFI: 0.94, TLI: 0.94, and RMSEA: 0.036. *R*^2^ Compulsions: 0.33.

The factor loadings of each latent indicator on its corresponding variables and the regression paths between latent variables of model 2 are displayed in **Figure 2**. All compulsive behavior indicators loaded highly on the latent factor (compulsions). SEM analysis of the proposed model, including control of depressive symptoms, indicated a good fit to the data: χ^2^(1160): 1782.38 (*p* < 0.001), CFI: 0.94, TLI: 0.94, and RMSEA: 0.036.

Similar to the results of model 1, the direct path between rumination and compulsion was no longer present after intrusion had been inserted as a mediating variable (β = -0.03, *p* = 0.78; without intrusions as a mediator: β = 0.43, *p* < 0.001). Accordingly, intrusion also fully mediated the relationship between rumination and compulsions. Rumination was highly associated with intrusion (β = 0.76), which was highly associated with compulsion (β = 0.60). The indirect effect from rumination to compulsions was 0.45 (*p* < .001, 95% CI [0.305, 0.612]).

This mediation model endured when symptoms of depression were added as a control variable (**Figure 2**; indirect effect from rumination to compulsions: 0.23, *p* < 0.001, 95% CI [0.111, 0.371]). Symptoms of depression were associated with rumination (β = 0.77) and intrusion (β = 0.52), but not with compulsion (β = -0.09) (**Figure 2**). This model explained 33% of the variance of compulsive behavior, 13% more than the same model without intrusions as a mediator (*R*^2^ = 0.20; χ^2^(933): 1472.45 (*p* < 0.001), CFI: 0.94, TLI: 0.94, and RMSEA: 0.037; using MLVM estimator).^1^

### Testing the Specificity of Results

To test the specificity of our results, we first checked the mediation when the independent variable (IV) and the mediator had switched their roles. For model 1 (IV: intrusions; mediator: rumination; DV: dysregulated behavior), the indirect effect no longer reached significance (*p* = 0.13; model fit: χ^2^(1999): 2790.02 (*p* < 0.001), CFI: 0.93, TLI: 0.93, and RMSEA: 0.031). In this alternative model, rumination showed no significant correlation with behavioral dysregulation (*r* = 0.16, *p* = 0.14). Also in model 2 (IV: intrusions; mediator: rumination; DV: compulsions), we found no significant indirect effect (*p* = 0.93; model fit: χ^2^(1160): 1782.38 (*p* < 0.001), CFI: 0.94, TLI: 0.94, RMSEA: 0.036). Rumination was not significantly correlated with compulsions (*r* = 0.01, *p* = 0.93). Additional analyses, in which the independent (IV) and dependent variables (DV) switched their roles, show lower indirect effects. In model 1 (IV: behavioral dysregulation, mediator: intrusions, DV: rumination) the indirect effect was 0.14 (*p* < 0.05) and in model 2 (IV: compulsions, mediator: intrusions, DV: rumination) 0.11 (*p* < 0.01).

Second, to check the specificity, we conducted SEM analyses with depressive symptoms as the mediator variable. We expected that rumination and depression would be highly correlated; however, depression might not play a mediating role. According to the ECM, negative affect might have a great impact. Beyond negative affect, depressive symptoms have a much broader scope, such as cognitive impairment, sleep disturbances, or low self-esteem. In model 1 (independent variable: rumination; mediator: depression; and dependent variable: behavioral dysregulation), indeed we found a high correlation between rumination and depression (*r* = 0.83; *p* < 0.001); however, there was no significant correlation between depressive symptoms and dysfunctional behavior (*r* = 0.09; *p* = 0.50), and no significant indirect effect (*p* = 0.50; model fit: χ^2^(2001): 2850.54 (*p* < 0.001), CFI: 0.93, TLI: 0.93, RMSEA: 0.032). Model 2 (independent variable: rumination; mediator: depression; and dependent variable: compulsions) showed significant correlations between rumination and depression (*r* = 0.77, *p* < 0.001), rumination and compulsions (*r* = 0.24, *p* < 0.05), and between depression and compulsions (*r* = 0.24, *p* < 0.05), and a significant indirect effect (*p* = 0.02; model fit: χ^2^(933): 1472.45 (*p* < 0.001), CFI: 0.94, TLI: 0.94, and RMSEA: 0.037). This result indicates a partial mediation. Including depression as a mediator, we found also a significant direct effect (*p* < 0.05); however, the relationship between rumination and compulsions was smaller (*r* = 0.24) compared to the model without depression (*r* = 0.43).

## Discussion

The current study aimed at replicating and extending the ECM by Selby et al. (2008) in a sample of the general population. A full mediating effect of intrusions on the relationship between rumination and a broad range of dysregulated behaviors was postulated and tested. To the best of our knowledge this study represents the first attempt to generalize the ECM to the realm of compulsions.

### Replication of the ECM

This replication of the ECM in a population-based sample underlines the important role of rumination concerning the onset of dysregulated and compulsive behaviors. The fact that all six, usually separately observed, dysregulated behaviors formed one latent factor that was highly correlated with rumination suggests that these behaviors share common aspects, mechanisms, and/or functions which might underline the described ECM. This idea is reinforced by the very similar results for a previously unexplored form of dysregulated behavior: compulsions. These findings imply that it might be worthwhile to consider rumination in the treatment of OCD and patients with other dysregulated behaviors. More functional ways of regulating aversive emotions and emotional thoughts, such as reappraisal or distraction, might prevent or at least weaken the vicious emotional cascade cycle described above.

### Intrusion as a Mediator between Rumination and Behavioral Dysregulation

Finding full mediation of intrusions in the relationship between rumination and dysregulated behaviors supports the hypothesis that rumination is particularly problematic because it fosters negative intrusive and uncontrollable emotional thoughts. Rumination presumably prolongs and intensifies the emotional reaction to an intrusive thought by focusing attention on it and triggering even more associated negative thoughts, leading to an EC. This may explain why people may develop dysregulated behaviors especially in times of high psychological “load” (e.g., more rumination and negative thoughts intensifying intrusions). It could also explain why those suffering from borderline personality disorder or posttraumatic stress disorder often develop chronic behavioral dysregulations. These patients permanently suffer from high levels of distress, tend to ruminate excessively, and are experience extremely aversive thoughts or intrusions (Ehlers et al., 2004; Law and Chapman, 2015).

Recently, Tuna and Bozo (2014) found that thought suppression is a relevant factor for emotional cascades. Using structural equation modeling, thought suppression correlated significantly with all observed dysfunctional behaviors (e.g., bulimia; *r*s ≥ 0.21), whereas rumination showed no significant or smaller correlations with dysfunctional behaviors (*r*s ≤ 0.17). Previous studies demonstrated that thought suppression can result in frequent intrusive thoughts (e.g., Salkovskis and Campbell, 1994). The present study extended the structural equation model by Tuna and Bozo (2014) by explicitly investigating intrusions as a mediating variable in the association between rumination and dysregulated behaviors.

Given their strong association, one might argue that intrusions and rumination actually reflect the same construct (e.g., negative repetitive thoughts). However, although intrusions and rumination were strongly associated, their correlation of *r* = 0.76 means that both constructs only share about 58% of common variance, so that 42% of variance is non-shared or unique. Beyond this empirical point, the two concepts differ on a theoretical level as well: Rumination refers to a certain *mode* of dealing with negative information, whereas the intrusion concept more strongly focuses on the (negative) *content* or product of thinking (or ruminating) itself (i.e., negative contents automatically entering working memory).

Unfortunately, there is still much to learn about the interplay between intrusions, rumination, and dysregulated behavior. On the one hand we know little about possible interplay between the content of intrusions and rumination, and on the other it remains unclear why these experiences may promote a specific type of dysregulated behavior. It might be true that differing content of rumination or intrusions may foster different types of dysregulated behaviors. For example, Law and Chapman (2015) found that anger and depressive rumination show different effects on negative cognitions. Depressive rumination compared to anger rumination elicited more self-blame. Regarding the temporal variation of rumination, the association between rumination and NSSI seems to be based on the instability of rumination (using the experience sampling method over 2 weeks; Selby et al., 2013). Furthermore, differences among various types of dysregulated behaviors were demonstrated in a recent study (Arbuthnott et al., 2015). After rumination, a patient history of NSSI was associated with higher levels of negative affect, whereas a history of eating disorder was associated with lower levels of positive affect.

### Intrusion as a Mediator between Rumination and Compulsions

We tested the extended ECM for five types of compulsions, assuming that the interplay of rumination and intrusion should comprise this intrusion-affected type of psychological problem. As expected, we found evidence of full mediation for intrusions. Some previous studies have also focused on the role of rumination and intrusion in OCD. Wahl et al. (2011b), for example, found that patients with OCD suffer not only from obsessive thoughts, but also from frequent ruminative thoughts. A between-group comparison showed that people with OCD and with a depressive disorder did not differ regarding the frequency of ruminative thoughts. In addition, the present study demonstrated that intrusions can play a mediating role in the relationship between rumination and compulsive behavior. This result supports the previous findings on the ECM and documents the informative value of the extended ECM for understanding one possible cause of compulsions.

### Specificity of Findings

In regard to statistically controlling for the current level of depressive symptoms, the proposed mediation model of dysregulated and compulsive behaviors remained significant. To further check the specificity of our findings, we tested alternative models: First, the independent and mediator variables were reversed. In both models (dependent variable: dysregulated behaviors/compulsions), rumination was not a significant mediator in the relationship between intrusions and behavioral dysregulation or compulsions, indicating the specificity of our results. So, in the emotional cascade, rumination may be associated with behavioral dysregulation through induced intrusions (rather than vice versa: intrusions through rumination). Although we cannot draw causal conclusions due to the cross-sectional nature of our study, models with rumination as independent variable, intrusions as mediator, and behavioral dysregulation/compulsions as dependent variable showed stronger indirect effects than models in which the independent and dependent variables switch their roles.

Second, we tested the mediating role of depression. In model 1 (independent variable: rumination; mediator: depression; dependent variable: behavioral dysregulation), depression was not a significant mediator in the association between rumination and dysregulated behavior. Depressive symptoms were not significantly associated with dysregulated behavior. In the emotional cascade, negative affect is assumed to play a relevant role in triggering dysregulated behavior. However, depressive symptoms have a much broader scope, such as cognitive impairment, sleep disturbances, or low self esteem. Furthermore, dysregulated behavior in this broad range (drinking to cope, anger-out, bulimia, social support/reassurance of the MIHT, and urgency, and reassurance of the DIRI) may not be specifically associated with depressive symptoms. In contrast, depressive symptoms partially mediated the relationship between rumination and compulsions. This finding is in line with previous research showing that rumination, depressive symptoms, and symptoms of OCDs are closely correlated (Menzies and de Silva, 2003; García-Soriano et al., 2014; Moradi et al., 2014). Despite this alternative model with a partial mediation of depression, all other alternative models found no significant mediation. In general, we tested theoretical models extending the ECM; however, we do not suggest that these are the only valid models, as additional mediators may exist. Furthermore, it should be acknowledged that compulsions (or OCD) only serve as an exemplar to study relevant processes within the framework of the emotional cascade model, and that the processes we have outlined might be of equal relevance in other clinical conditions (e.g., eating disorders).

### Limitations and Further Implications

Although the current study has important strengths, such as use of a population-based sample and a sufficiently large and powered sample for structural equation modeling, there were some limitations to note. The first is the response bias in our postal questionnaire study. We do not know which characteristics may differentiate people returning the questionnaire from those deciding not to participate (e.g., higher education or young/middle age). Although the sample was similar to the overall German population with respect to important demographic features, the sample is not totally representative of this population (e.g., regarding the age distribution) and it might be that more subtle differences between responders and non-responders, such as introspection, may influence the results.

Another limitation of this study is the application of a non-clinical sample with somewhat lower levels of dysregulated and compulsive behaviors (**Tables 1** and **2**). Yet, despite this limitation, the ECM still fit the data well. Further investigations should test the ECM in clinical populations, especially in those in which intrusions play an important role (e.g., OCD, general anxiety disorder, and post-traumatic stress disorder).

Furthermore, only six dysregulated and five compulsive behaviors were investigated. Although they formed one second-order factor each, supporting the idea that the ECM might explain many kinds of dysregulated behaviors, the generalization of the ECM to other behaviors is still uncertain.

Regarding the psychometric quality of the measures, we acknowledge that the two items of the DIRI had poor internal consistency (α = 0.47) and that the use of only two items for SEM analyses is not recommended. We decided to use only these two items of the DIRI because the other two items are nearly identical (e.g., “Do you frequently seek reassurance from the people you feel close to as to whether they really care about you?” and “Do you find yourself often asking the people you feel close to how they truly feel about you?”).

As mentioned above, despite the high correlation between rumination and intrusions (*r* = 0.76), the two constructs are not redundant. However, there may have been issues associated with multicollinearity between the measures of rumination and intrusion. Multicollinearity can cause more inexact estimations of the predictors, an overfitting in the model or the full mediation effect in our model. The correlation between intrusions and rumination is indeed high; however, correlations above *r* = 0.80 are classified as questionable (Backhaus et al., 2003). Furthermore, the model including intrusions as a mediator explained 14% more variance of behavioral dysregulation compared to the model without intrusive thoughts. So, despite the high correlation, the two constructs may make a unique contribution. In general, however, we cannot completely rule out biases due to potential multicollinearity.

Another limitation to consider is the temporal discrepancy between our measures of lifetime habitual means of emotion regulation, compulsions, behavioral dysregulation tendencies, and intrusions, as related to our measure of depressive symptoms, which were assessed over the prior 2 weeks. Future studies should use experimental or ambulant monitoring methods (e.g., Selby et al., 2013) to investigate the relation of simultaneously appearing intrusions and rumination, and the latency until behavioral dysregulation takes place. In addition, a closer examination of the intrusions would show whether varying contents have a different influence on the probability of an ECM occurring and the specific type of behavioral dysregulation.

Finally, due to our cross-sectional design we cannot draw definite causal conclusions. In the context of the second study (*N* = 175), we primarily checked the test-retest reliability of our measures. Longitudinal analyses of our models were not computed (e.g., rumination at time 1 and behavioral dysregulation at time 2), because these analyses (with this complexity and the used estimation procedure WLSMV) require larger sample sizes (*N* ≥ 200–250) and study 2 did not include all the necessary measures. Additionally, our cross-sectional study does not answer the question of whether the triggering of dysregulated behaviors by rumination and intrusions lasts only for a short while or may lead to a long-lasting process of ECs following each other. Investigating the duration and progress of these interactive effects may also reveal influencing factors that could end the vicious cycle of rumination, intrusions, negative affect, and behavioral dysregulation. Investigating the temporal conditions of ECM would allow the development of successful psychotherapeutic interventions. Further studies using experimental procedures (e.g., mood induction in the laboratory), as well as methods of ecological momentary assessment (Fahrenberg et al., 2007; Selby et al., 2013) to gain a more fine-grained understanding of the exact temporal dynamics of the ECM, appear promising.

## Conclusion

The finding that intrusions fully mediated the relation between rumination and six types of dysregulated and five types of compulsive behaviors has implications for psychotherapy, although we cannot draw final causal conclusions. Up to now rumination has normally not been specifically addressed in disorders in which intrusions are a symptom or focus of treatment (e.g., OCD), nor are intrusions commonly considered in the psychotherapy of typical “rumination disorders” such as depression. It would seem worthwhile to help patients to become aware of their usage of rumination and to learn to engage in more functional emotion regulation strategies instead of using dysregulated behaviors. Researchers and clinicians should also develop more adaptive ways to deal with intrusions and to decrease their general level of distress, to reduce the probability and negativity of intrusions.

## Author Contributions

Conception and design of the work was chiefly done by MW and NV, and acquisition by NV. Analysis done by SJ and all authors (SJ, NV, ES, and MW) interpreted the data. SJ wrote the article and all the authors did critical revision. Final version to be published was approved by SJ, NV, ES, and MW. All authors agreed to be accountable for all aspects of the work in ensuring that questions related to the accuracy or integrity of any part of the work are appropriately investigated and resolved.

## Conflict of Interest Statement

The authors declare that the research was conducted in the absence of any commercial or financial relationships that could be construed as a potential conflict of interest.

## Footnotes

1 In alternatively tested moderator models, the interaction term of intrusions and rumination did not explain further variance underlining that the mediational model explains the data best.
